# Denitrifying Bacteria Active in Woodchip Bioreactors at Low-Temperature Conditions

**DOI:** 10.3389/fmicb.2019.00635

**Published:** 2019-04-02

**Authors:** Jeonghwan Jang, Emily L. Anderson, Rodney T. Venterea, Michael J. Sadowsky, Carl J. Rosen, Gary W. Feyereisen, Satoshi Ishii

**Affiliations:** ^1^The BioTechnology Institute, University of Minnesota, Saint Paul, MN, United States; ^2^Department of Soil, Water, and Climate, University of Minnesota, Saint Paul, MN, United States; ^3^Soil and Water Management Research Unit, Agricultural Research Service, United States Department of Agriculture, Saint Paul, MN, United States

**Keywords:** denitrification, nitrate removal, woodchip bioreactor, drainage, *Cellulomonas*

## Abstract

Woodchip bioreactor technology removes nitrate from agricultural subsurface drainage by using denitrifying microorganisms. Although woodchip bioreactors have demonstrated success in many field locations, low water temperature can significantly limit bioreactor efficiency and performance. To improve bioreactor performance, it is important to identify the microbes responsible for nitrate removal at low temperature conditions. Therefore, in this study, we identified and characterized denitrifiers active at low-temperature conditions by using culture-independent and -dependent approaches. By comparative 16S rRNA (gene) analysis and culture isolation technique, *Pseudomonas* spp., *Polaromonas* spp., and *Cellulomonas* spp. were identified as being important bacteria responsible for denitrification in woodchip bioreactor microcosms at relatively low temperature conditions (15°C). Genome analysis of *Cellulomonas* sp. strain WB94 confirmed the presence of nitrite reductase gene *nirK*. Transcription levels of this *nirK* were significantly higher in the denitrifying microcosms than in the non-denitrifying microcosms. Strain WB94 was also capable of degrading cellulose and other complex polysaccharides. Taken together, our results suggest that *Cellulomonas* sp. denitrifiers could degrade woodchips to provide carbon source and electron donors to themselves and other denitrifiers in woodchip bioreactors at low-temperature conditions. By inoculating these denitrifiers (i.e., bioaugmentation), it might be possible to increase the nitrate removal rate of woodchip bioreactors at low-temperature conditions.

## Introduction

Nitrogen (N) and phosphorus (P) are the most important nutrients in fertilizers for agriculture. While some of them are taken up by plants or adsorbed to minerals or organic matter, a proportion of the nutrients can be leached from agricultural fields into rivers, lakes, and oceans, causing eutrophication (USEPA, 2008; MPCA, 2014). Agricultural runoff water from the Upper Midwest States is considered a major cause of the hypoxic zone, also known as the dead zone, in the Gulf of Mexico (USEPA, 2008).

Large amounts of nutrients are released from agricultural fields through subsurface (tile) drainage, which is installed to improve soil conditions for root growth and soil trafficability for timely planting and harvesting (Bhattarai et al., 2005). While artificial subsurface drainage has increased agricultural productivity, it has also increased the amount of nutrients, especially nitrate, released from fields into surrounding waterways (Gentry et al., 1998).

One approach to remove nitrate from subsurface drainage water is to install denitrifying bioreactors at the end of the drainpipes before water is discharged to ditches or streams (Warneke et al., 2011). A woodchip bioreactor is a subsurface trench filled with woodchips through which drainage water passes. The woodchips provide a carbon and energy source to denitrifying microorganisms (Schipper et al., 2010; Ghane et al., 2015). Although woodchip bioreactors have demonstrated success in nitrate removal in many field locations (Christianson et al., 2012a), low water temperature during the cold seasons significantly limits bioreactor performance (Christianson et al., 2012b; David et al., 2016), which is likely related to the low metabolic activity of denitrifying microorganisms at low temperatures. In addition to cold temperatures (<5°C) in winter and early spring, water temperature usually ranges only from 10 to 20°C throughout the remainder of the year (Ghane et al., 2015), implying that microorganisms adapted to low temperatures might play important roles for nitrate removal more generally within woodchip bioreactors.

Previous woodchip bioreactor research has focused largely on the hydrology and engineering aspects of the system (Greenan et al., 2009; Moorman et al., 2010; Ghane et al., 2015; Lepine et al., 2016; Sharrer et al., 2016), although microorganisms play key roles in the technology. There have been a few reports on the microbial communities in woodchip bioreactors by using quantitative PCR (qPCR) or restriction fragment length polymorphism (RFLP) targeting denitrification functional genes (Warneke et al., 2011; Hathaway et al., 2015; Healy et al., 2015; Porter et al., 2015). However, it is still unclear which specific microorganisms are responsible for nitrate removal in woodchip bioreactors. This is partly due to difficulties in identifying denitrifying microorganisms. Denitrifying ability is sporadically distributed among taxonomically diverse groups of bacteria, archaea and fungi (Knowles, 1982; Zumft, 1997; Ishii et al., 2009). Both denitrifying and non-denitrifying strains can be present in the same genus; therefore, it is difficult to identify denitrifying organisms based on taxonomic information alone. In addition, denitrifiers in different taxa can have almost identical denitrification functional gene sequences (Philippot, 2002; Jones et al., 2008; Ishii et al., 2011). Therefore, it is also difficult to identify microorganisms based on the denitrification functional gene sequence information.

To overcome this issue, comparative 16S rRNA gene sequencing analyses has been successfully used to identify denitrifying microorganisms (Ishii et al., 2009). In this approach, microbial communities under different conditions (i.e., denitrification and non-denitrification conditions) are compared to identify microorganisms that increased their abundance under denitrification conditions. This is based on the assumption that microorganisms that grow or become more active under denitrification conditions are most likely denitrifiers. This assumption was proven feasible because most denitrifiers identified by the comparative 16S rRNA gene sequencing analysis (Ishii et al., 2009) were later isolated and confirmed as bona fide denitrifiers (Ishii et al., 2011).

In this study, we used the comparative 16S rRNA (gene) sequencing analysis to identify denitrifying microorganisms active at the relatively low temperature conditions found in a woodchip bioreactor. We used both DNA and RNA to identify total and metabolically active microorganisms, respectively (Gremion et al., 2003; Yoshida et al., 2012). In addition, we isolated denitrifying microorganisms from the same woodchip samples. By characterizing these microorganisms, it may be possible to develop a strategy to enhance denitrification activity of woodchip bioreactors using bioaugmentation and biostimulation strategies. Consequently, the objective of this study was to (i) identify denitrifiers active at low-temperature conditions by comparative 16S rRNA (gene) analysis, (ii) isolate low temperature-adapted denitrifiers by culture method, and (iii) characterize these denitrifying strains.

## Materials and Methods

### Woodchip Bioreactor Microcosms

Woodchip samples were collected from a 4-year-old field bioreactor near Willmar, MN, on October 2, 2014. Detailed description of the site was reported previously (Ghane et al., 2018). In brief, the woodchip bioreactor (106.4 m long and 1.51–1.84 m wide) was originally installed in November 2010. The bioreactor had received tile drain water from nearby corn and corn-soybean fields. The 4-year-old woodchips were excavated by using a backhoe, and stored in tightly sealed sterile plastic bags at 4°C until used. The 4-year-old woodchips contained 24–32% cellulose, 15–25% hemicellulose, and 27–31% lignin (Ghane et al., 2018).

For microcosm experiments, 5 g of woodchips were placed in 210-mL vials, and mixed with 5 mL of synthetic tile drain water (see Supplementary Table S1 for chemical composition) supplemented with or without 3.57 mM nitrate and/or 3.95 mM acetate. Nitrate concentration of 3.57 mM was used to provide enough electron acceptor for denitrification during incubation. This level of nitrate has also been observed in tile drain water in the field (Gamble et al., 2018). The concentration of acetate used provided a C/N molar ratio of around 2.0, which was previously reported as the minimum value needed to reduce almost all of the nitrate to dinitrogen (N_2_) gas (Her and Huang, 1995). The vial headspace was replaced with anoxic gas according to standard method (Tiedje, 1994). While N_2_ and acetylene (C_2_H_2_) in a 90:10 ratio was used to measure N_2_O gas accumulated during incubation (i.e., acetylene inhibition method; (Tiedje, 1994), N_2_ gas was used for microbial analysis. In both cases, microcosms were incubated at 15°C for up to 48 h. Four treatments were prepared by using this microcosm setup: (i) WINA, woodchip microcosm incubated with nitrate and acetate; (ii) WIN, woodchip microcosm incubated with nitrate but without acetate; (iii) WIA, woodchip microcosm incubated with acetate but without nitrate; and (iv) WI, woodchip microcosm incubated with neither nitrate nor acetate. In addition, woodchip samples without incubation (= treatment W) were collected when microcosms were prepared. The treatment W functioned as a no-incubation control (=0-h control).

To determine the occurrence of denitrification, microcosms were incubated in triplicate at 15°C with the vial headspace containing 10% C_2_H_2_. The concentration of N_2_O in the head space was measured at 0, 4, 8, 12, 24, 36, and 48 h after incubation, by using a gas chromatograph (GC) (Model 5890, Hewlett-Packard/Agilent Technologies) equipped with an electron capture detector and PoraPak Q column (Sigma-Aldrich) as previously described (Maharjan and Venterea, 2013). The water samples were collected from the microcosms at 0, 12, 24, 36, and 48 h after incubation, and filtered through 0.22-μm-pore membranes to analyze the concentrations of dissolved organic carbon (DOC). DOC was measured by using an Elementar vario TOC Select in TIC/TC/TNb mode.

### RNA and DNA Extractions

For RNA and DNA extractions, a different set of woodchip microcosms were prepared with the vial headspace filled with 100% N_2_. Nine vials were prepared for each treatment (a total of 36 vials). The microcosms were incubated as described above. Three microcosms were sacrificed for each treatment 24, 36, and 48 h after incubation, and the RNA and DNA were extracted from woodchip samples (2 g) collected from each of the microcosms. In addition, RNA and DNA were extracted from woodchip samples (*n* = 3) without incubation (treatment W).

RNA and DNA were extracted by using a PowerSoil RNA Isolation kit (MOBIO, Carlsbad, CA, United States) and RNA PowerSoil DNA Elution Accessory kit (MOBIO, Carlsbad, CA, United States), respectively, according to the manufacturer’s instructions. For the extracted RNA samples, possible genomic DNA residue was removed using Turbo DNA free kit (Ambion, Austin, TX, United States). No DNA contamination in the resulting RNA samples was confirmed by PCR targeting the 16S rRNA gene as described previously (Ishii et al., 2016). Complementary DNA (cDNA) was synthesized from the RNA samples (200 ng) by using PrimeScript RT Reagent kit (Takara Bio, Mountain View, CA, United States) and random hexamers according to the manufacturer’s instructions.

### Microbial Community Analysis

Thirty nine DNA and cDNA samples shown in Supplementary Table S2 were individually used to amplify the V4 region of the 16S rRNA gene and 16S rRNA using the 515F–806R primer set, respectively, as described previously (Caporaso et al., 2012). Resulting amplicons were purified and used to prepare Illumina sequencing libraries with the TruSeq kit (Illumina, San Diego, CA, United States). Paired-end sequencing reaction was done using a MiSeq platform (Illumina) with V3 chemistry (300-bp read length) at the University of Minnesota Genomics Center (Minneapolis, MN, United States).

The paired-end raw read data were assembled, quality-filtered and trimmed using NINJA-SHI7 (Al-Ghalith et al., 2018), which is a fastq-to-combined-fasta processing pipeline. The assembled sequences were clustered into OTUs at 97% sequence similarity using NINJA-OPS (Al-Ghalith et al., 2016), which is a complete OTU-picking pipeline with advantage of the Burrows-Wheeler alignment using BowTie2. The resulting OTU tables, in sparse BIOM 1.0 format, were used for further statistical analyses done using QIIME 1.9.1 (Caporaso et al., 2010). Taxonomic assignment of the OTUs were done using the Greengenes 97 reference data set (McDonald et al., 2011).

### Culture-Independent Identification of Denitrifiers

Microbes responsive to the denitrification-inducing conditions (i.e., denitrifiers) were identified by comparing the microbial communities in denitrification-inducing conditions (i.e., treatments WINA and WIN) and those in non-denitrification conditions (i.e., treatments WIA and WI). The following steps were used for this analysis: (1) OTUs showing more than 1% relative abundance in at least one of the triplicate samples were chosen as major and represented microbial taxa; (2) OTUs showing a significant difference between the three sample types (i.e., microcosms incubated with nitrate [treatments WINA and WIN], microcosms incubated without nitrate [treatments WIA and WI], and no incubation control [treatment W]) were identified by analysis of variance (ANOVA) test (FDR *p* < 0.05); and (3) OTUs that satisfied both steps 1 and 2 were visualized by heatmap analysis done with the Bray-Curtis distance indices. The OTU heatmaps were created by the heatmap.2 and vegdist subroutines within the gplots and vegan packages, respectably, for R.

### Isolation and Identification of Denitrifiers

Denitrifying bacteria were also directly isolated from the woodchip samples collected from the same field bioreactor near Willmar, MN. In brief, 1 g of the woodchip sample, stored at 4°C as described above, was mixed with phosphate buffered saline (PBS, pH 7.4). The woodchip suspension was then spread-plated onto R2A agar plates, supplemented with 5 mM nitrate and 10 mM acetate (R2A-NA). The plates were incubated under anaerobic conditions, by using AnaeroPack system (Mitsubishi Gas Chemical), at 15°C for 1 to 2 weeks. Colonies were picked and restreaked onto new R2A-NA agar plates to obtain well-isolated single colonies.

The ability of the strains to denitrify was examined by using the acetylene inhibition assay (Tiedje, 1994). In brief, fresh cell cultures (300 μl) were inoculated into R2A-NA broth (10 ml) in 27 ml test tubes. After replacing the air phase with Ar:C_2_H_2_ (90:10) gas, the test tubes were incubated at 30°C. The temperature of 30°C was used for the acetylene inhibition assay because all strains isolated grew faster at 30°C than at 15°C, and therefore, they likely produced more N_2_O at 30°C in 2 weeks. After 2-week incubation, gas samples were taken via a gastight syringe and analyzed for N_2_O production by GC as described above. In addition, liquid samples were collected and analyzed for nitrate, nitrite and ammonium concentrations using the SEAL AA3 HR AutoAnalyzer. Strains that reduced ≥40% nitrate, converted <10% of nitrate to ammonium, and produced significant amount of N_2_O (>100 ppm) were considered as denitrifiers. The GC system used in this study was too sensitive, and the upper quantification limit was often exceeded. Therefore, we could not calculate the percentage of nitrate reduced to N_2_O.

Genomic DNA were isolated by heating cells in 100 μl 0.05 M NaOH at 95°C for 15 min (Ashida et al., 2010). After centrifugation, the supernatant was diluted 10-fold in MilliQ water, and used for PCR to amplify the 16S rRNA gene. The reaction mixture (50 μl) contained 1× Ex Taq buffer (Takara Bio, Otsu, Japan), 0.2 μM of each primer [m-27F and m-1492R; (Tyson et al., 2004)], 0.2 mM of each dNTP, 1 U of Ex Taq DNA polymerase (Takara Bio), and 2 μl of DNA template. PCR was carried out using a VeritiTM Thermal Cyclers (Life Technologies) and the following conditions: initial annealing at 95°C for 5 min, followed by 30 cycles of 95°C for 30 s, 55°C for 30 s and 72°C for 90 s, and one cycle of 72°C for 7 min. Amplification was confirmed by using agarose gel electrophoresis. The PCR products were purified using AccuPrep PCR Purification Kit (Bioneer) and then quantitated using PicoGreen dsDNA quantitation assay (Thermo Fisher Scientific). The purified PCR products were sequenced by the Sanger method using a 3730*xl* DNA Analyzer (Applied Biosystems) at the University of Minnesota Genomics Center. The forward (m-27F) and reverse (m-1492R) reads were assembled using the phred, phrap, consed software (Ewing et al., 1998). Strain identity was determined by using a Naïve Bayesian classifier (Wang et al., 2007).

### Whole Genome Sequencing

*Cellulomonas* sp. strain WB94 was selected for genome sequencing since this bacterium increased its relative abundance under denitrifying conditions based on the comparative 16S rRNA sequencing analysis. Genomic DNA was extracted from pure cell cultures using PowerSoil DNA Isolation Kit (MOBIO) according to the manufacturer’s instructions. Sequencing libraries were prepared using the PacBio SMRT kit (Pacific Biosciences), and the genome was analyzed by using the PacBio RS II platform (Pacific Biosciences). Extracted DNA was used to generate a SMRTbell library (20 kbp insert) which was sequenced at the Mayo Clinic’s Molecular Biology Core (Rochester, MN, United States). After quality filtering, reads were assembled *de novo* using the hierarchical genome assembly process (HGAP3) in the SMRT Link portal (v 2.3.0). Genome annotation was done using the NCBI Prokaryotic Genome Annotation Pipeline (Tatusova et al., 2016). Average Nucleotide Identity (ANI) values were calculated using JSpecies (Richter and Rosselló-Móra, 2009).

### Transcription Analysis of the *Cellulomonas nirK*

Primers WB94_nirK_F (5′-AGACGCTGTGGACCTACAAC-3′) and WB94_nirK_R (5′-CGACGAACTGGTACGTCAAC-3′) were designed based on the genome sequence of *Cellulomonas* sp. WB94 and used to amplify *nirK* transcripts of this strain. Specificity of this primer set was verified by using NCBI Primer-Blast.^1^ The reaction mixture for qPCR (10 μL) contained 1× SYBR Premix ExTaq ROX plus (Takara Bio), 0.2 μM each primer, and 2 μL of cDNA samples. The qPCR was performed using StepOnePlus Real-Time PCR System v. 2.3 (Applied Biosystem) with the following conditions: 95°C for 30 s, followed by 45 cycles of 95°C for 5 s, 60°C, and 83°C for 30 s. Melting curve analysis and agarose gel electrophoresis were done to confirm correct amplification of the PCR products. In addition to the *Cellulomonas nirK*, the quantity of 16S rRNA was measured by qPCR with Eub338 (5′-ACTCCTACGGGAGGCAGCAG-3′) and Eub518 (5′-ATTACCGCGGCTGCTGG-3′) primers (Muyzer et al., 1993). Levels of *nirK* transcripts were normalized using the quantity of 16S rRNA.

### Statistical Analyses

The PAST software was used to perform one-way ANOVA test to analyze statistical significance in the quantitative data obtained in microcosm treatments (Hammer et al., 2001).

### Nucleotide Sequence Accession Numbers

The 16S rRNA amplicon sequences were deposited to the Short Read Archive under accession number SRP149200. The 16S rRNA gene sequences of the isolated strains and the whole genome sequence of strain WB94 have been deposited in the DDBJ/EMBL/GenBank databases under accession numbers MH196452–MH196472 and NZ_QEES00000000, respectively.

## Results

### Occurrence of Denitrification in the Microcosms

To identify denitrifiers active in the woodchip bioreactors at relatively low-temperature conditions, we established a series of reproducible woodchip bioreactor microcosms to evaluate the following four treatments: (i) WINA, woodchip microcosm incubated with nitrate and acetate; (ii) WIN, woodchip microcosm incubated with nitrate but without acetate; (iii) WIA, woodchip microcosm incubated with acetate but without nitrate; and (iv) WI, woodchip microcosm incubated without nitrate and acetate. The microcosms were incubated at 15°C at anoxic conditions. In addition, no-incubation control (treatment W) was also prepared. In this study, 15°C was used as a low temperature conditions because 15°C is generally considered as the optimum growth temperature for psychrophiles (Morita, 1975). Although water temperature in field woodchip bioreactors can become <5°C, it also becomes >15°C for more than 2 months (Feyereisen et al., 2018). Therefore, 15°C is a temperature that can be seen in the field bioreactors in MN. This temperature is still considered low as compared with other denitrification studies (e.g., 30°C; Tiedje, 1994; Ishii et al., 2009; Tago et al., 2011). In our preliminary experiments, we also used different temperatures (4 and 10°C) to isolate more-cold-adapted denitrifying bacteria; however, no colony appeared in 2 weeks (data not shown), and therefore, we did not use these temperatures for the microcosm experiments.

**Figure 1:**
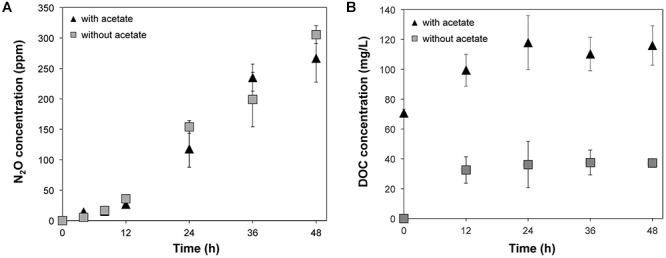
**(A)** N_2_O production and **(B)** DOC concentrations from the microcosms supplemented with nitrate (i.e., treatments WINA and WIN) during 48-h incubation. N_2_O production was not observed from the microcosms without nitrate addition (i.e., treatments WIA and WI): 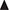, Microcosms incubated with nitrate and acetate (i.e., treatment WINA) and 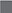, microcosms incubated with nitrate only (i.e., treatment WIN).

Accumulation of N_2_O was observed in the microcosms supplemented with nitrate regardless of the addition of acetate (Figure 1A), suggesting that denitrification occurred in these conditions (i.e., treatments WINA and WIN). The N_2_O concentrations were not significantly different (*p* = 0.7084 by ANOVA) between WINA and WIN treatments, suggesting that enough carbon and electrons were present in the woodchips to promote denitrification. Nitrous oxide was not detected in the microcosms without addition of nitrate, indicating that denitrification did not occur in these conditions (i.e., treatments WIA, and WI). Concentrations of N_2_O in the microcosms incubated ≥24 h were significantly larger (*p* < 0.05 by ANOVA) than those incubated ≤12 h, suggesting that denitrification activity greatly increased after 12 h.

Concentrations of dissolved organic carbon (DOC) significantly increased within 12 h of incubation (*p* < 0.05 by ANOVA) in the microcosms of the treatments WINA and WIN (Figure 1B). Since denitrification occurred in these two conditions at a similar level, DOC produced as a result of the degradation of woodchips most likely provided enough carbon and electrons to denitrifiers.

### Microbial Communities in the Microcosms

RNA and DNA were extracted from the microcosms after 0-, 24-, 36-, and 48-h incubations, and used for the microbial community analyses (Supplementary Table S2). A total of 2,731,477 and 3,741,963 sequence reads were obtained from 39 DNA to 39 cDNA samples, respectively. The number of sequences per sample ranged from 28,609 to 115,611 and from 21,530 to 181,499 for DNA and cDNA samples, respectively. Numbers of sequences were normalized to the smallest number among the DNA and cDNA samples by random subsampling for further downstream analyses. The subsampled sequences provided sufficient resolution of the microbial communities, as indicated by Good’s coverage ranging from 0.962 to 0.979 (Supplementary Table S3) and by analysis of rarefaction curves (Supplementary Figure S1).

**Figure 2:**
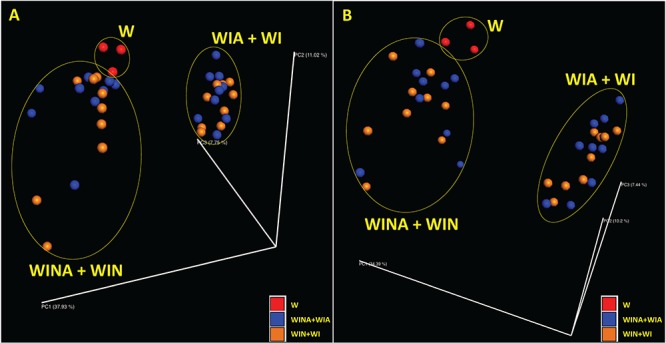
Principal coordinate analysis (PCoA) plots showing β diversity between microbial communities for **(A)** DNA and **(B)** cDNA samples. The β diversity was calculated using Bray-Curtis dissimilarity. Red, no-incubation control (i.e., treatment W); blue, microcosms incubated with acetate (i.e., treatments WINA and WIN); and orange, microcosms incubated without acetate (i.e., treatments WIA and WI). Microbial communities in the microcosms incubated with nitrate (i.e., treatments WINA and WIN) were clustered together.

Supplementary Table S3 also shows species richness estimated by observed operational taxonomic units (OTUs) and Chao1 index, and species diversity represented by Shannon and Simpson indices, for microbial community in each DNA and cDNA sample. These diversity indices were significantly lower in the microbial communities from the woodchips incubated with nitrate (i.e., treatments WINA and WIN) than those from the woodchips incubated without nitrate (i.e., treatments WIA and WI) (*p* < 0.05 by ANOVA). However, no significant differences were observed between the microbial communities from the woodchips incubated with acetate (i.e., treatments WINA and WIA) and those from the woodchips incubated without acetate (i.e., treatments WIN and WI) (*p* > 0.05 by ANOVA). This suggested that α diversity in a microbial community is more influenced by the nitrate addition than by the addition of acetate. Moreover, the addition of nitrate influenced the β diversity as well. Microbial communities in the microcosms incubated with nitrate (i.e., treatments WINA and WIN) clustered differently from those in the microcosms incubated without nitrate (i.e., treatments WIA and WI) based on principal coordinate analysis (PCoA) plots with Bray-Curtis dissimilarity for both DNA (Figure 2A) and cDNA (Figure 2B) samples. No clustering of microbial communities was observed by acetate addition (Figure 2A,B), suggesting that the addition of external carbon source such as acetate had minimal impact on α and β diversities of the microbial communities. Microbial communities were relatively similar between denitrifying microcosms (i.e., treatments WINA and WIN) and no-incubation controls (i.e., treatment W). No-incubation control is the woodchip samples collected from the field bioreactor, in which denitrification actively occurred. Therefore, microbial communities in treatment W might represent denitrifying communities, similar to those in treatments 2A and 2B.

### Microbial Taxa Responsive to Denitrification

**Figure 3:**
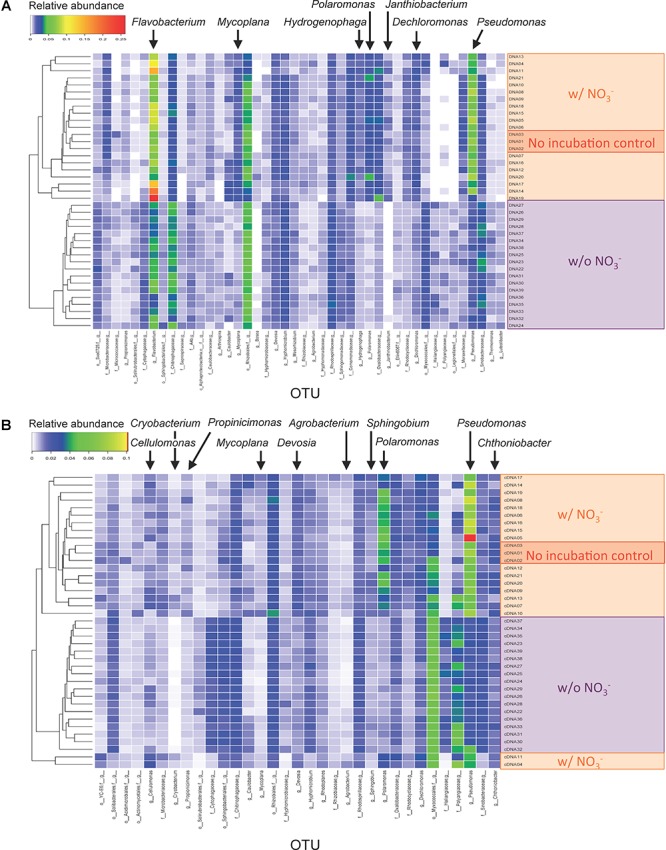
Heatmaps showing relative abundance of sequence reads in operational taxonomic units (OTUs) for **(A)** DNA and **(B)** cDNA samples. Only OTUs that showed different abundance between incubation conditions are shown. Assigned genus names are shown for the OTUs that showed significant differences between the three sample types (i.e., microcosms incubated with nitrate [treatments WINA and WIN], microcosms incubated without nitrate [treatments WIA and WI], and no-incubation control [treatment W]) by analysis of variance (ANOVA) test. For detailed sample information, see Supplementary Table S2.

The OTUs responsive to the denitrification-inducing conditions were identified by comparative 16S rRNA (gene) analysis (Figure 3). OTUs (266 and 232) were identified as having different relative abundance between three sample types (i.e., microcosms incubated with nitrate [treatments WINA and WIN], microcosms incubated without nitrate [treatments WIA and WI], and no incubation control [treatment W]) by ANOVA test (FDR *p* < 0.05), for DNA and cDNA samples, respectively. Among the 266 OTUs identified in the DNA analysis, those classified to the genera *Dechloromonas*, *Flavobacterium*, *Hydrogenophaga*, *Janthinobacterium*, *Mycoplana*, *Polaromonas*, and *Pseudomonas* were significantly more abundant in microcosms incubated with nitrate addition than those incubated without nitrate (Figure 3A). Among the 232 OTUs identified in the RNA (cDNA) analysis, those classified to the genera *Agrobacterium*, *Cellulomonas*, *Cryobacterium*, *Devosia*, *Mycoplana*, *Polaromonas*, *Propionicimonas*, *Pseudomonas*, and *Sphingobium* were significantly more abundant in microcosms incubated with nitrate addition than those incubated without nitrate (Figure 3B). Since these OTUs increased their abundance in response to denitrifying conditions, they are most likely denitrifiers or nitrate reducers. *Pseudomonas* and *Polaromonas* were significantly more abundant in denitrifying conditions than non-denitrifying conditions for both DNA and RNA samples, indicating that they were active and rapidly growing denitrifiers in the woodchip samples at relatively cold conditions (15°C).

### Denitrifiers Isolated From the Woodchip Bioreactors

A total of 104 strains were isolated from the woodchip bioreactor samples incubated with nitrate and acetate at 15°C under anoxic conditions. Among those, 21 isolates were identified as nitrate-reducing and N_2_O-producing bacteria by the acetylene inhibition assay. Most isolates belonged to three genera: *Cellulomonas* (3 strains), *Clostridium* (14 strains), and *Microvirgula* (3 strains). Since bacteria reducing nitrate to ammonium (i.e., dissimilatory nitrate reduction to ammonium; DNRA) can also produce N_2_O in the acetylene inhibition test (Tiedje, 1994), we measured concentrations of nitrate and ammonium to discriminate DNRA bacteria from denitrifying bacteria. Bacteria that reduced >10% of nitrate to ammonium were considered as DNRA bacteria. By this analysis, four strains of *Clostridium* spp. and one *Cellulomonas* spp. strains remained as denitrifying bacteria (Supplementary Table S4).

**Figure 4:**
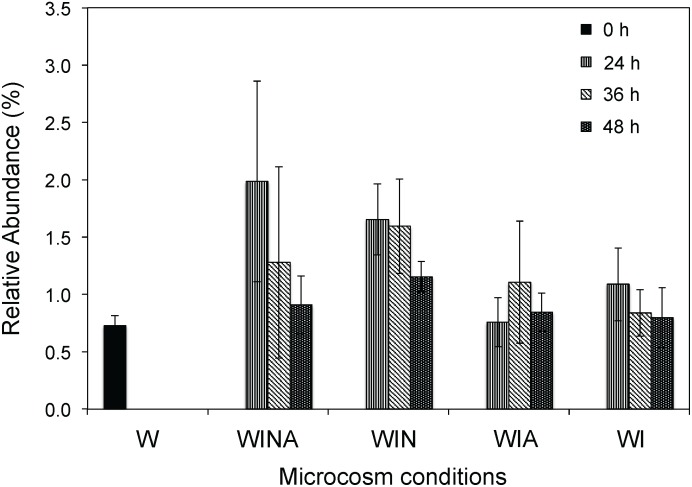
Relative abundance (%) of *Cellulomonas* rRNA in the sequencing libraries. Mean ± SD (*n* = 3) is shown. W, no-incubation control; WINA, woodchip microcosms incubated with nitrate and acetate additions; WIN, woodchip microcosms incubated with nitrate addition; WIA, woodchip microcosms incubated with acetate addition; WI, woodchip microcosms incubated without any additives.

The genus *Cellulomonas* was commonly detected by both culture-dependent and –independent approaches. Compared with the control microcosms, the abundance of members of the genus *Cellulomonas* significantly increased in the RNA samples collected from the denitrifying microcosms (*p* < 0.05 by ANOVA), but not in those collected from the non-denitrifying microcosms (*p* = 0.33 by ANOVA) (Figure 4). Taken together, these results suggested that *Cellulomonas* spp. strains are likely one of the most active denitrifying bacteria in the woodchip bioreactor samples. Although all *Cellulomonas* spp. strains were isolated at 15°C, these strains grew faster at 30°C under denitrifying conditions, suggesting that they have broad growth temperature range, but are not psychrophilic.

### Whole Genome Analysis of *Cellulomonas* sp. Strain WB94

The presence of denitrification functional genes could not be detected by PCR with commonly used primers. To identify genes related to denitrification and cellulose degradation, we sequenced the genome of *Cellulomonas* sp. strain WB94 by using the PacBio platform. The genome of strain WB94 (accession number NZ_QEES00000000) was represented by seven contigs, with a total genome size of 3,868,980 bp and mole% G + C content ranging from 0.70 to 0.73% (Supplementary Table S5). The genome contained 3,387 protein-coding sequences (CDS), 137 pseudogenes, 46 tRNAs, six rRNAs (two rRNA operons), and three non-coding RNAs. The average nucleotide identity (ANI) between the genomes of strain WB94 and *Cellulomonas cellasea* DSM 20118 were 98%, which is greater than the cutoff value for species discrimination (95–96%) (Goris et al., 2007; Richter and Rosselló-Móra, 2009). Therefore, strain WB94 most likely belonged to *Cellulomonas cellasea*.

**Figure 5:**
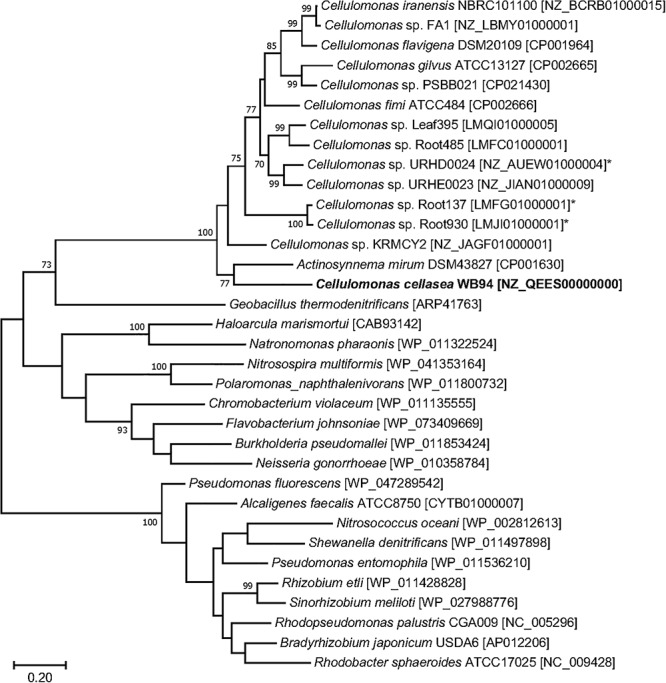
Phylogenetic tree generated based on the deduced NirK sequences using the maximum likelihood method. GenBank accession numbers are shown in square brackets. Bootstrap values (%) were generated from 1000 replicates, and the values >70% are shown. Branch lengths correspond to sequence differences as indicated by the scale bar.

The genome of strain WB94 harbored the nitrate reductase genes *narGHJI* and *napA* as well as the dissimilatory NO-forming nitrite reductase gene *nirK* (Supplementary Table S6), suggesting that strain WB94 can reduce nitrate to nitrite and to nitric oxide. The deduced NirK amino acid sequence was most closely related to the NirK from *Actinosynnema mirum* DSM43827 [CP001630], but was also similar to those from other *Cellulomonas* species (>57% identity) (Figure 5). Other denitrification-related genes were not found on the genome. The genome also contained the genes *nirBD* encoding assimilatory NAD(P)H-dependent nitrite reductase which reduces nitrite to ammonium in the cytoplasm, suggesting that strain WB94 can use nitrate and nitrite as a *N* source. The genome also contained genes related to the biodegradation of complex polysaccharides, including cellulose, xylan, starch and glycogen (Supplementary Table S6).

### Potential Ecological Role of *Cellulomonas* spp. in the Woodchip Bioreactors

**Figure 6:**
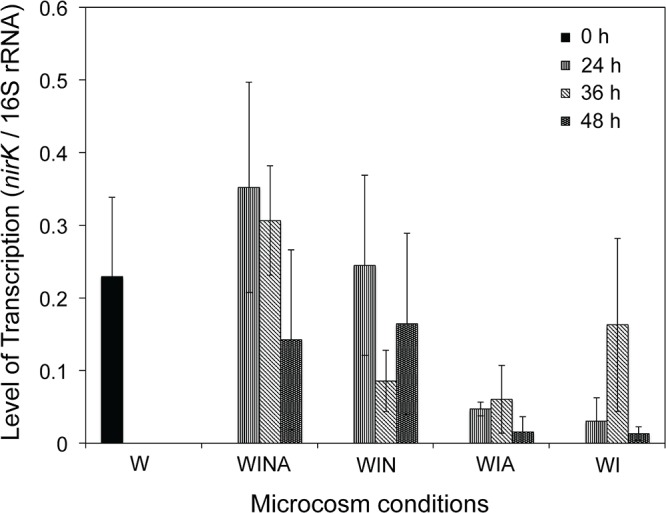
Transcription level of *Cellulomonas nirK* in the woodchip microcosms. Transcription levels were normalized by the amount of the 16S rRNA. Mean ± SD (*n* = 3) is shown. W, no-incubation control; WINA, woodchip microcosms incubated with nitrate and acetate additions; WIN, woodchip microcosms incubated with nitrate addition; WIA, woodchip microcosms incubated with acetate addition; WI, woodchip microcosms incubated without any additives.

We measured the transcription levels of *Cellulomonas nirK* (Figure 6) to verify the denitrification activity of *Cellulomonas* spp. in the woodchip bioreactor microcosm. Levels of *nirK* transcription were significantly higher in the denitrifying microcosms (WINA and WIN) than in non-denitrifying microcosms (WIA and WI) (*p* < 0.01 by ANOVA). Interestingly, however, the *nirK* transcription levels in the no incubation controls (W) were also significantly greater than those in the non-denitrifying microcosms (*p* < 0.01 by ANOVA) but not significantly different from those in the denitrifying microcosms (*p* = 0.87). No-incubation control is the woodchip samples collected from actively denitrifying field bioreactor; therefore, *Cellulomonas* sp. denitrifiers might have been active in this condition.

The biodegradation of cellulose by *Cellulomonas* sp. strain WB94 was also verified by using the cellulase assay (data not shown), suggesting that they could degrade cellulose in the woodchips.

## Discussion

While woodchip bioreactor technology is a promising approach to reduce nutrient loading from agricultural fields to surrounding and downstream water bodies (Christianson et al., 2012a), limited research has been done to identify denitrifiers in these bioreactors. In this study, we used both culture-dependent and –independent approaches to identify denitrifying microorganisms active at relatively low temperature conditions in a woodchip bioreactor.

Similar amounts of N_2_O were produced from triplicate woodchip bioreactor microcosms, suggesting that denitrification occurred reproducibly in the microcosms used in this study. The amount of N_2_O significantly increased after 12-h incubation at 15°C, suggesting that the microorganisms actively performed denitrification after 12 h. Addition of acetate did not increase the amount of N_2_O produced, indicating that carbon and electrons were not limited. This lack of improvement in nitrate removal rate with acetate addition to woodchips is in contrast to a recent laboratory column study that showed enhanced performance at 15 and 5°C (Roser et al., 2018). Others have shown that woodchip nitrate removal performance is negatively affected as the woodchips age (Robertson, 2010; David et al., 2016). Bioreactors with fresh woodchips showed better *N* removal than those with aged woodchips probably because fresh woodchips contain more readily available C (Robertson, 2010; David et al., 2016). Thus, in the current study, even though 4-year-old woodchips were used, there was still enough C available for denitrification from the woodchips that the addition of readily available C (acetate) did not enhance denitrification rate. This difference could be methodological or attributed to a robust microbial community in this experiment.

A comparative 16S rRNA (gene) sequencing approach was used to identify denitrifying microorganisms. A similar approach was successfully used to identify denitrifying bacteria in rice paddy soils (Ishii et al., 2009). While this previous study used conventional clone library analysis with >1,000 clones/library, here we used Illumina MiSeq high-throughput sequencing technology with >20,000 reads/sample. As a result, we sequenced enough DNA to cover the majority of microorganisms in the samples. In addition, Ishii et al. (2009) only used DNA samples, whereas here we sequenced the 16S rRNA (gene) from both DNA and RNA to identify total and metabolically active microorganisms, respectively. Sequencing 16S rRNA was previously shown useful to detect metabolically active microorganisms (Gremion et al., 2003; Yoshida et al., 2012) because more ribosomes are present in metabolically active cells than resting or starved cells (Nomura et al., 1984). Microbial community structures were different between DNA- and RNA-based analyses, similar to previous studies (Gentile et al., 2006; Moeseneder Markus et al., 2006; Lanzén et al., 2011), suggesting that only parts of the microbial populations were active in the environments.

Several genera were identified as potential denitrifying bacteria. *Pseudomonas* spp. and *Polaromonas* spp. were commonly detected both by DNA- and RNA-based analyses. The genus *Pseudomonas* includes well-studied denitrifying strains such as *Pseudomonas stutzeri* strain ZoBell and *Pseudomonas aeruginosa* strain PAO1 and is reported to be one of the most active denitrifiers in natural environments (Knowles, 1982). In addition, some strains such as *P. aeruginosa* strain PKE117 and *Pseudomonas putida* strain mt-2 strains are reported to have strong extracellular lignin peroxidase activities to degrade woodchips (Shui Yang et al., 2007; Ahmad et al., 2010), suggesting that *Pseudomonas* spp. could perform denitrification and use woodchips as a C source. *Polaromonas* species are also known to be psychrophiles with temperature optima 4–12°C (Irgens et al., 1996). Nitrate reduction of the *Polaromonas* strains have been reported (Mattes et al., 2008; Margesin et al., 2012), and a complete set of denitrification functional genes is present in the draft genome of *Polaromonas glacialis* R3-9 strain (GenBank accessions NZ_KL448323 and NZ_KL448327) (Wang et al., 2014), suggesting that *Polaromonas* spp. could perform denitrification at low temperature conditions.

Some genera were detected by the DNA- or the RNA-based analyses, but not by both methods. For example, the genera *Cellulomonas*, *Cryobacterium*, *Propionicimonas*, *Devosia*, *Agrobacterium*, and *Sphingobium* were detected only by the RNA-based analysis. The difference may be due to the growth rates of bacteria. Metabolically active cells may also replicate and increase their rRNA gene copies in the environment; however, there is a time lag between metabolic activity and genome replication (Rolfe et al., 2012). Therefore, active but slow-growing bacteria may not always be detected by the DNA-based analysis.

*Cellulomonas* spp. were commonly detected by both culture-independent analysis and culture-dependent isolation methods. Other genera identified as denitrifying bacteria by the culture-independent analysis were not obtained by our isolation method, probably due to the bias caused by the medium used (i.e., R2A-NA). Growth media can largely influence results of bacterial isolation (Davis et al., 2005).

Although denitrification by *Cellulomonas* strains has not been reported thus far, an incomplete set of denitrification functional genes (e.g., *narG* and *nirK*) is present in several genomes of the *Cellulomonas* sp. strains (GenBank accessions CP001964, CP002665, CP002666, and CP021430). Our *Celluomonas* sp. strain WB94 also possessed denitrification functional genes, including *narG* and *nirK*, and was able to reduce nitrate. The *nirK* of strain WB94 was similar to those from other *Cellulomonas* species. Transcription levels of the *Cellulomonas nirK* were significantly higher in the denitrifying microcosms than the non-denitrifying microcosms, suggesting that *Cellulomonas* strains were actively involved in denitrification process in woodchip bioreactors. Genes responsible for nitric oxide (NO) reductase were not found on the genome, likely due to incomplete genome assembly of this genome. Since WB94 produced N_2_O by the acetylene inhibition assay, this strain should have NO reductase on its genome. Genome completion and further data mining is necessary to identify the NO reductase of this strain.

*Cellulomonas* spp. are also well known for their ability to use endoglucanases and exoglucanases to degrade cellulose (Thayer et al., 1984). Our strain, *Cellulomonas* sp. strain WB94, also had the ability to degrade cellulose. In addition, various genes related to the biodegradation of complex polysaccharides were found on the genome of strain WB94. These results suggest that *Cellulomonas* spp. could play an important role in nitrate reduction as well as degradation of woodchips.

## Conclusion

In conclusion, based on a series of culture-independent and –dependent analyses, we identified *Pseudomonas* spp., *Polaromonas* spp., and *Cellulomonas* spp. as being important bacteria responsible for denitrification in woodchip bioreactor microcosms at relatively cold temperature conditions. This is the first report of clearly identifying denitrifiers that are active in relatively cold woodchip bioreactor conditions. Since *Cellulomonas* spp. identified in this study can also degrade cellulose and other complex polysaccharides, they may provide a C source and electron donors to themselves and other denitrifiers in woodchip bioreactors. Inoculating these denitrifiers (i.e., bioaugmentation) could increase the nitrate removal rate of woodchip bioreactors at low temperature conditions (Feyereisen et al., 2018). Further characterization of these strains such as growth and substrate utilization rates under varying conditions would help design and optimize bioreactor operating conditions.

This microcosm-based study was designed to mimic field conditions of *N* concentration and temperature, but the study’s batch method differed from the continuous flow of field bioreactors. To examine if the denitrifiers identified in this study are also active in the field conditions, it is necessary to analyze samples collected from the field, which should be done in the future.

## Data Availability

The datasets generated for this study can be found in DDBJ/EMBL/GenBank databases, SRP149200, MH196452–MH196472, and NZ_QEES00000000.

## Author Contributions

JJ and SI designed the research. JJ, EA, and RV performed the experiments. JJ, EA, RV, and SI analyzed the data. JJ, EA, RV, MS, CR, GF, and SI wrote the manuscript.

## Conflict of Interest Statement

The authors declare that the research was conducted in the absence of any commercial or financial relationships that could be construed as a potential conflict of interest.

## Footnotes

1 https://www.ncbi.nlm.nih.gov/tools/primer-blast/
