# Supplementary material for: Denitrifying Bacteria Active in Woodchip Bioreactors at Low-Temperature Conditions
Source: Front Microbiol. 2019 Apr 2;10:635. doi: 10.3389/fmicb.2019.00635 (PMC6454037; doi:10.3389/fmicb.2019.00635)
Supplement: Supplementary file 1 [file Data_Sheet_1.PDF]

Supplemental Materials

Denitrifying Bacteria Active in Woodchip Bioreactors at Low-Temperature Conditions

Jeonghwan Jang[1], Emily Anderson[2], Rodney T. Venterea[2,3], Michael J. Sadowsky[1,2], Carl Rosen[2], Gary W. Feyereisen[3], Satoshi Ishii[1,2,*]

[1]BioTechnology Institute, University of Minnesota, St. Paul, MN
[2]Department of Soil, Water, and Climate, University of Minnesota, St. Paul, MN
[3]USDA-ARS, Soil and Water Management Research Unit, St. Paul, MN

* Corresponding author: Satoshi Ishii, ishi0040@umn.edu

This file contains 1 figure and 6 tables.

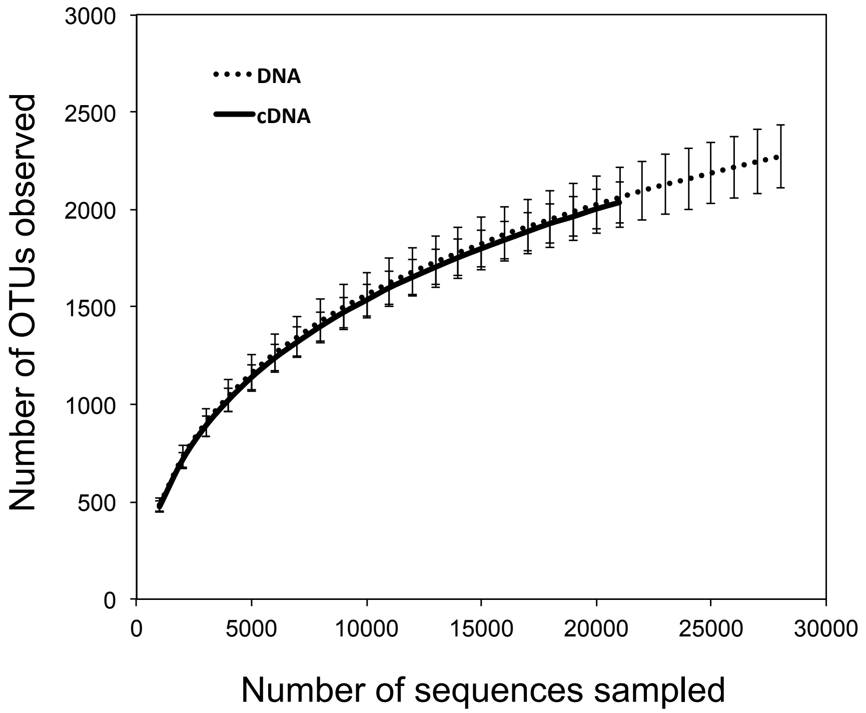

Figure S1. Rarefaction curve generated based on the 16S rRNA (gene) sequences obtained in this study. Total sequence reads were normalized to 28,609 and 21,530 reads per library for DNA and cDNA samples, respectively.

Table S1. Composition of the synthetic agricultural wastewater

Chemical	Concentration (mg/L)
$CaCl_2$	220.5
$MgCl_2.6H_2O$	421.5
KH_2PO_4	1.3
Na_2SO_4	10.4
H_3BO_3	0.1
$FeSO_4.7H_2O$	0.625
$CuSO_4.5H_2O$	0.0775
$MnSO_4.H_2O$	0.025
$ZnSO_4.7H_2O$	0.1

Table S2. Samples prepared for the MiSeq 16S rRNA (gene) sequencing and *nirK* qPCR analyses.

Sample ID	Treatment ID	Supplement		Incubation time (h)	Sample type
		Nitrate	Acetate		
DNA01	W	−	−	0	DNA
DNA02	W	−	−	0	DNA
DNA03	W	−	−	0	DNA
DNA04	WINA	+	+	24	DNA
DNA05	WINA	+	+	24	DNA
DNA06	WINA	+	+	24	DNA
DNA07	WIN	+	−	24	DNA
DNA08	WIN	+	−	24	DNA
DNA09	WIN	+	−	24	DNA
DNA10	WINA	+	+	36	DNA
DNA11	WINA	+	+	36	DNA
DNA12	WINA	+	+	36	DNA
DNA13	WIN	+	−	36	DNA
DNA14	WIN	+	−	36	DNA
DNA15	WIN	+	−	36	DNA
DNA16	WINA	+	+	48	DNA
DNA17	WINA	+	+	48	DNA
DNA18	WINA	+	+	48	DNA
DNA19	WIN	+	−	48	DNA
DNA20	WIN	+	−	48	DNA
DNA21	WIN	+	−	48	DNA
DNA22	WIA	−	+	24	DNA
DNA23	WIA	−	+	24	DNA
DNA24	WIA	−	+	24	DNA
DNA25	WI	−	−	24	DNA
DNA26	WI	−	−	24	DNA
DNA27	WI	−	−	24	DNA
DNA28	WIA	−	+	36	DNA
DNA29	WIA	−	+	36	DNA
DNA30	WIA	−	+	36	DNA
DNA31	WI	−	−	36	DNA
DNA32	WI	−	−	36	DNA
DNA33	WI	−	−	36	DNA
DNA34	WIA	−	+	48	DNA
DNA35	WIA	−	+	48	DNA
DNA36	WIA	−	+	48	DNA
DNA37	WI	−	−	48	DNA
DNA38	WI	−	−	48	DNA
DNA39	WI	−	−	48	DNA

Table S2 (continued)

Sample ID	Treatment ID	Supplement		Incubation time (h)	Sample type
		Nitrate	Acetate		
cDNA01	W	−	−	0	RNA (cDNA)
cDNA02	W	−	−	0	RNA (cDNA)
cDNA03	W	−	−	0	RNA (cDNA)
cDNA04	WINA	+	+	24	RNA (cDNA)
cDNA05	WINA	+	+	24	RNA (cDNA)
cDNA06	WINA	+	+	24	RNA (cDNA)
cDNA07	WIN	+	−	24	RNA (cDNA)
cDNA08	WIN	+	−	24	RNA (cDNA)
cDNA09	WIN	+	−	24	RNA (cDNA)
cDNA10	WINA	+	+	36	RNA (cDNA)
cDNA11	WINA	+	+	36	RNA (cDNA)
cDNA12	WINA	+	+	36	RNA (cDNA)
cDNA13	WIN	+	−	36	RNA (cDNA)
cDNA14	WIN	+	−	36	RNA (cDNA)
cDNA15	WIN	+	−	36	RNA (cDNA)
cDNA16	WINA	+	+	48	RNA (cDNA)
cDNA17	WINA	+	+	48	RNA (cDNA)
cDNA18	WINA	+	+	48	RNA (cDNA)
cDNA19	WIN	+	−	48	RNA (cDNA)
cDNA20	WIN	+	−	48	RNA (cDNA)
cDNA21	WIN	+	−	48	RNA (cDNA)
cDNA22	WIA	−	+	24	RNA (cDNA)
cDNA23	WIA	−	+	24	RNA (cDNA)
cDNA24	WIA	−	+	24	RNA (cDNA)
cDNA25	WI	−	−	24	RNA (cDNA)
cDNA26	WI	−	−	24	RNA (cDNA)
cDNA27	WI	−	−	24	RNA (cDNA)
cDNA28	WIA	−	+	36	RNA (cDNA)
cDNA29	WIA	−	+	36	RNA (cDNA)
cDNA30	WIA	−	+	36	RNA (cDNA)
cDNA31	WI	−	−	36	RNA (cDNA)
cDNA32	WI	−	−	36	RNA (cDNA)
cDNA33	WI	−	−	36	RNA (cDNA)
cDNA34	WIA	−	+	48	RNA (cDNA)
cDNA35	WIA	−	+	48	RNA (cDNA)
cDNA36	WIA	−	+	48	RNA (cDNA)
cDNA37	WI	−	−	48	RNA (cDNA)
cDNA38	WI	−	−	48	RNA (cDNA)
cDNA39	WI	−	−	48	RNA (cDNA)

Table S3. Richness and α diversity indices of the microbial communities in the woodchip microcosms. Total sequence reads were normalized to 28,609 and 21,530 reads per library for DNA and cDNA samples, respectively.

Sample ID	Good's coverage	Richness		Diversity	
		Observed OTUs	Chao1	Shannon	Simpson
DNA01	0.973	2222	3171.4	9.093	0.995
DNA02	0.973	2320	3174.3	9.263	0.996
DNA03	0.973	2289	3203.8	9.257	0.996
DNA04	0.974	2162	3133.3	9.071	0.995
DNA05	0.973	2285	3126.7	9.100	0.995
DNA06	0.974	2215	3066.8	9.111	0.995
DNA07	0.973	2212	3186.4	9.232	0.996
DNA08	0.973	2265	3190.2	9.162	0.995
DNA09	0.975	2100	2954.7	9.038	0.995
DNA10	0.974	2220	3078.0	9.212	0.996
DNA11	0.977	2020	2719.1	8.865	0.992
DNA12	0.975	2167	3012.5	9.181	0.996
DNA13	0.973	2266	3169.2	9.284	0.996
DNA14	0.976	1984	2876.5	8.404	0.986
DNA15	0.973	2264	3115.8	9.160	0.995
DNA16	0.972	2264	3294.6	9.168	0.995
DNA17	0.974	2190	3055.3	8.843	0.991
DNA18	0.971	2303	3463.6	9.222	0.995
DNA19	0.979	1839	2531.2	7.988	0.974
DNA20	0.976	2030	2791.6	8.878	0.994
DNA21	0.974	2188	3066.2	9.162	0.995
DNA22	0.973	2432	3326.9	9.472	0.996
DNA23	0.974	2332	3170.4	9.384	0.996
DNA24	0.970	2594	3691.2	9.643	0.997
DNA25	0.973	2389	3323.0	9.487	0.997
DNA26	0.970	2588	3559.2	9.624	0.997
DNA27	0.972	2403	3401.2	9.495	0.997
DNA28	0.971	2488	3467.5	9.567	0.997
DNA29	0.969	2564	3716.6	9.539	0.997
DNA30	0.972	2422	3402.6	9.446	0.996
DNA31	0.974	2370	3223.7	9.488	0.997
DNA32	0.975	2262	3011.6	9.383	0.997
DNA33	0.974	2326	3170.2	9.432	0.997
DNA34	0.974	2396	3241.4	9.508	0.997
DNA35	0.975	2317	3113.7	9.457	0.997
DNA36	0.974	2357	3166.1	9.475	0.997
DNA37	0.972	2500	3336.3	9.554	0.997
DNA38	0.972	2430	3386.5	9.487	0.997
DNA39	0.975	2394	3232.1	9.583	0.997

Table S3 (continued)

Sample ID	Good's coverage	Richness		Diversity	
		Observed OTUs	Chao1	Shannon	Simpson
cDNA01	0.965	2088	3101.0	9.198	0.996
cDNA02	0.965	2082	3080.0	9.152	0.995
cDNA03	0.964	2124	3088.4	9.204	0.996
cDNA04	0.971	1821	2532.3	8.576	0.990
cDNA05	0.967	1997	2817.2	8.923	0.994
cDNA06	0.967	2000	2827.2	9.051	0.995
cDNA07	0.969	1954	2698.5	9.036	0.995
cDNA08	0.966	2019	2894.3	9.045	0.995
cDNA09	0.969	1912	2663.6	9.024	0.995
cDNA10	0.967	2018	2848.3	9.124	0.996
cDNA11	0.966	1938	2922.6	8.771	0.992
cDNA12	0.967	2056	2862.6	9.199	0.996
cDNA13	0.966	1965	2954.4	8.927	0.993
cDNA14	0.970	1752	2614.0	8.534	0.990
cDNA15	0.965	1996	2920.4	9.035	0.995
cDNA16	0.967	1968	2829.5	9.036	0.995
cDNA17	0.967	1966	2896.3	8.961	0.994
cDNA18	0.966	2012	2937.3	9.094	0.995
cDNA19	0.967	1960	2815.2	9.016	0.995
cDNA20	0.969	1929	2751.6	9.090	0.995
cDNA21	0.966	2009	2931.0	9.117	0.995
cDNA22	0.966	2169	3019.9	9.239	0.994
cDNA23	0.966	2146	2980.5	9.276	0.995
cDNA24	0.962	2258	3265.6	9.407	0.996
cDNA25	0.967	2119	2832.1	9.269	0.995
cDNA26	0.964	2208	3147.2	9.373	0.996
cDNA27	0.965	2072	3134.6	9.218	0.995
cDNA28	0.966	2135	3003.2	9.347	0.996
cDNA29	0.963	2243	3145.0	9.333	0.995
cDNA30	0.966	2093	2915.7	9.051	0.992
cDNA31	0.964	2107	3136.8	9.161	0.994
cDNA32	0.968	1993	2825.1	9.123	0.994
cDNA33	0.967	2031	2942.4	9.154	0.994
cDNA34	0.964	2185	3238.6	9.401	0.996
cDNA35	0.966	2122	2962.4	9.294	0.995
cDNA36	0.967	2108	2953.5	9.278	0.995
cDNA37	0.963	2210	3177.3	9.420	0.996
cDNA38	0.965	2227	3080.0	9.417	0.996
cDNA39	0.966	2170	3074.3	9.335	0.995

Table S4. Nitrate reducing and denitrifying strains obtained in this study. Strains shown in bold reduced ≥40% nitrate, converted <10% of nitrate to ammonium, and produced significant amount of N_2O (>100 ppm), and therefore, were considered as denitrifiers.

Isolate ID	Proportion of N converted to ammonium (%)	Nitrate reduced (%)	N_2O produced (ppm)	Identification (genus)
WB17	40.9	98.3	1401.1	*Microvirgula*
WB18	44.9	98.3	1479.3	*Microvirgula*
WB19	4.1	BDL	63.1	*Clostridium*
WB21	5.8	BDL	224.0	*Clostridium*
WB22	42.1	98.4	1496.5	*Microvirgula*
WB23	7.0	BDL	9.5	*Clostridium*
WB24.2	7.3	BDL	7.1	*Clostridium*
WB26	BDL	33.5	2.5	*Clostridium*
WB29	BDL	32.0	1.3	*Clostridium*
WB39	7.1	39.4	68.8	*Clostridium*
WB40	2.0	3.8	5.1	*Clostridium*
WB49	6.8	39.6	103.2	*Clostridium*
**WB53**	**0.8**	**58.2**	**843.6**	***Clostridium***
**WB66**	**5.0**	**45.1**	**112.1**	***Clostridium***
**WB76**	**BDL**	**44.4**	**147.0**	***Clostridium***
**WB80**	**5.9**	**49.8**	**603.2**	***Clostridium***
WB81	4.1	47.5	0.3	*Clostridium*
WB91	7.0	38.7	169.7	*Desulfobacterium*
**WB94**	**6.5**	**49.2**	**116.0**	***Cellulomonas***
WB102	2.5	60.3	BDL	*Cellulomonas*
WB104	73.1	29.1	994.9	*Cellulomonas*

Table S5. Summary of the sequenced genome of *Cellulomonas* sp. strain WB94.

Contig No.	Accession number	Size (bp)	GC content (%)
0	NZ_QEES01000002.1	2,780,765	71.9
1	NZ_QEES01000005.1	329,035	70.2
2	NZ_QEES01000001.1	235,040	70.3
3	NZ_QEES01000007.1	151,423	71.1
4	NZ_QEES01000004.1	162,011	72.2
5	NZ_QEES01000003.1	157,415	72.7
6	NZ_QEES01000006.1	53,291	71.9

Table S6. Genes associated with denitrification or polysaccharide catabolism identified on the genome of *Cellulomonas* sp. strain WB94.

	Function	Gene	Locus_tag	Product
Denitrification	Nitrate Reduction	*narI*	DDP54_03075	respiratory nitrate reductase subunit gamma
		narJ	DDP54_03080	nitrate reductase molybdenum cofactor assembly chaperone
		narH	DDP54_03085	nitrate reductase subunit beta
		narG	DDP54_03090	nitrate reductase subunit alpha
	Nitrite reduction	*nirD*	DDP54_03030	nitrite reductase [NAD(P)H] small subunit
		nirB	DDP54_03035	nitrite reductase [NAD(P)H]
			DDP54_03150	nitrite reductase [NAD(P)H]
		nirK	DDP54_17680	NO-forming nitrite reductase
Polysaccharide catabolism	Cellulose degradation		DDP54_00625	endoglucanase
		malQ	DDP54_01650	4-alpha-glucanotransferase
			DDP54_0629	1,3-beta-glucanase
		malQ	DDP54_17500	4-alpha-glucanotransferase
			DDP54_09215	cellobiose phosphorylase
	Xylan degradation		DDP54_00375	1,4-beta-xylanase
	Starch/glycogen degradation		DDP54_12980	alpha-amylase
			DDP54_13300	alpha-amylase
			DDP54_12980	alpha-amylase
			DDP54_13300	alpha-amylase
			DDP54_15400	glucoamylase
			DDP54_1540	glucoamylase
